# Case report: Senescence as mechanism of resistance to Pembrolizumab in a Lymphoma patient who failed CD19-Targeted CAR-T cell therapy

**DOI:** 10.3389/fimmu.2022.994731

**Published:** 2022-10-06

**Authors:** Serena De Matteis, Beatrice Casadei, Ginevra Lolli, Michele Dicataldo, Francesco Barbato, Elisa Dan, Andrea Paccagnella, Barbara Sinigaglia, Clara Bertuzzi, Annalisa Arcari, Luca Zazzeroni, Patrizia Bernuzzi, Noemi Laprovitera, Gianluca Storci, Salvatore Nicola Bertuccio, Manuela Ferracin, Massimiliano Bonafè, Pier Luigi Zinzani, Francesca Bonifazi

**Affiliations:** ^1^ IRCCS Azienda Ospedaliero-Universitaria di Bologna, Bologna, Italy; ^2^ Department of Experimental, Diagnostic and Specialty Medicine (DIMES), University of Bologna, Bologna, Italy; ^3^ Hematology and Bone Marrow Transplant Unit, “Guglielmo da Saliceto” Hospital, Piacenza, Italy; ^4^ Department of Medical and Surgical Sciences (DIMEC), University of Bologna, Bologna, Italy

**Keywords:** lymphoma, senescence, exhaustion, chimeric antigen receptor (CAR T), pembrolizumab, resistance

## Abstract

**Background:**

T cells engineered to target CD19 antigen on neoplastic B cells represent the most striking example of CAR-T cell therapy. The success rate of this therapy is affected by several limitations: target antigen loss, and/or acquisition of a senescent/exhausted phenotype by CAR and non-CAR T cells.

**Case presentation:**

We report on a patient affected by refractory Diffuse Large B-cell Lymphoma who was resistant to CAR T-cell therapy and to two cycles post CAR-T of pembrolizumab (PBZ) due to the evolution into a B-cell Hodgkin-like lymphoma. Owing to the CD30 expression and the Hodgkin-like phenotype, the patient was ultimately treated with Brentuximab-Vedotin and finally underwent remission. Upon PBZ treatment, 100% of circulating CAR-T^+^ cells showed a persistent CD8^+^ senescent/exhausted phenotype, while an increase in the percentage of senescent cells was found in the non-CAR CD8^+^ T cells compartment.

**Conclusions:**

PBZ is not able to reinvigorate exhausted CAR^+^ T cells and to confer durable clinical response. We hypothesize that the phenomenon is due to the senescent phenotype of CAR^+^ T cells, which did not allow PBZ-induced reactivation and proliferative rescue. The phenomenon, together with the loss of CAR-T target CD19 and the shift of non-CAR CD8^+^ T cells towards a senescent phenotype likely contributed to set up an immune landscape with poor antitumor capacity.

## Background

Anti-CD19 antigen CAR T-cell therapy represents a successful approach for many subtypes of non-Hodgkin lymphoma, resulting in favorable clinical response, with varying extent and timing (1, 2). Nevertheless, the success of CD19-Targeted CAR-T cell therapy is limited by the loss of target antigen due to tumor evolution, clonal selection and the acquisition of a senescent and/or exhausted phenotype by CAR and non-CAR T cells (3–6). The capability of PBZ to reinvigorate exhausted CAR-T cells was reported in Lymphoma patients who failed CAR-T cell therapy, being the response to the drug highly heterogenous and even absent in some patients (7). Here, we report on a patient affected by refractory Diffuse Large B-cell Lymphoma (DLBCL) who underwent CD19-targeted CAR-T cell (Tisagenlecleucel) therapy and, due to the evolution into a B-cell Hodgkin-like lymphoma, received two additional cycles of PBZ, which turned out to be ineffective in controlling the disease.

## Case presentation

**Figure 1:**
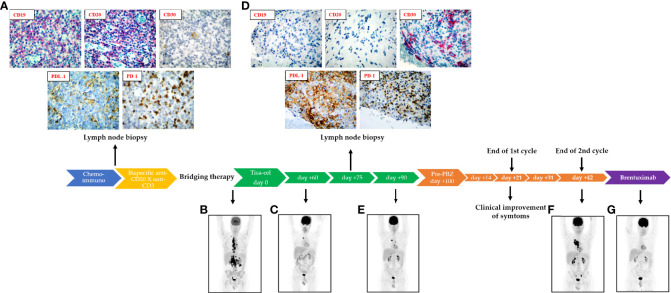
Clinical course. **(A)** Clinical timeline with immunohistochemical evaluation of lymph node biopsy at pre-bispecific (anti-CD20Xanti-CD3) antibody stage, that shows CD19 and CD20 positivity, CD30 negativity, the expression of PD-L1 in the neoplastic population and PD-1 in the tumor microenvironment. Relevant PET/TC scans at: **(B)** pre-CAR-T cell therapy, **(C)** day +60 after CAR-T cell infusion. **(D)** Immunohistochemical evaluation of lymph node biopsy at: pre-PBZ administration stage, that shows CD19 and CD20 negativity, CD30 positivity, the expression of PD-L1 in the neoplastic population and PD-1 in the tumor microenvironment. Relevant PET/TC scans: **(E)** at day +90 after CAR-T cell therapy, **(F)** at day +42 following PBZ treatment, **(G)** after the 4^th^ cycle of Brentuximab.

In November 2019, a 37-years-old male was diagnosed with a grade 2 Follicular Lymphoma, with stage IV/high risk, according to the Follicular Lymphoma International Prognostic Index, with bulky disease at onset. The patient was refractory to anti-CD20 (Obinutuzumab)-CHOP (Cyclophosphamide, Doxorubicin, Vincristine and Prednisone) and anti-CD20 (Rituximab)-DHAP (Cisplatin, high-dose Cytarabine and Prednisone). A post-therapy lymph node biopsy revealed a diagnosis of DLBCL, germinal center B-cell (GCB) like, with the expression of CD19 and CD20 pan-B-cell antigens (**Figure 1A**). The patient underwent bispecific anti-CD20 X anti-CD3 antibody (RGN1979), which however turned out to be ineffective in controlling the disease. Due to the anti-CD20 refractoriness above reported, the patient was referred to our Center to proceed with Tisagenlecleucel therapy. The fluorodeoxyglucose (FDG) positron emission tomography (PET)/CT scan at screening showed multiple supra and sub diaphragmatic adenopathies and an extensive skeletal involvement (**Figure 1B**). After lymphocyte-apheresis, the Ifosfamide-containing bridging therapy yielded a partial disease control. After lymphodepletion (LD) with fludarabine (25 mg/m^2^) and cyclophosphamide (250 mg/m^2^), a single infusion with Tisagenlecleucel was administered. Forty-eight hours after infusion, the patient experienced a grade 1 cytokine release syndrome, which spontaneously resolved after 24 hours. The PET/CT scan at day+60 (**Figure 1C**) revealed a stable disease with a response in some lymph nodes, but higher FDG-avidity in others, in comparison to the PET/CT performed at day+30. At day +75 after CAR-T cell infusion, the patient underwent a PET-guided biopsy of the abdominal lymph nodes (**Figure 1D**). The diagnosis was of Hodgkin-like B cell lymphoma, with a defective B-phenotype due to the lack of CD20 and CD19 expression (**Figure 1D**). Moreover, a population of atypical CD30 positive lymphocytes, a strong PD-L1 expression in tumor cells, concomitant to the up-regulation of PD-1 expression in the tumor microenvironment were observed (**Figure 1D**, see **Figure 1A** for comparison). The PET/CT scan at day +90 (**Figure 1E**) revealed a progression disease and at day +100, the patient received the first dose of PBZ (200 mg flat dose) showing a rapid resolution of symptoms the patient complained before starting therapy, such as abdominal pain, serotine fever and fatigue. Nevertheless, two months later, the patient experienced a clinical progression confirmed by PET/CT scan that showed FDG-uptake in multiple lymph nodes, right anonymous vein thrombosis and bilateral pleural effusion (**Figure 1F**). Due to the CD30 expression and the Hodgkin-like phenotype, the patient was finally treated by Brentuximab-Vedotin. The schedule was 1.8 mg/kg every 21 days. The PET/CT scan performed after the 4^th^ cycle of therapy in October 2021 showed a complete response (**Figure 1G**).

**Figure 2:**
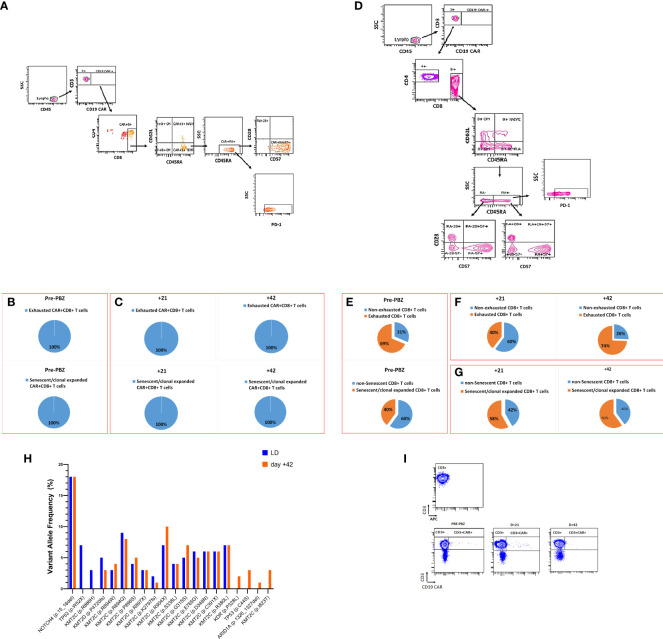
Flow cytometric analysis of peripheral blood CAR^+^ and CAR-non expressing T cell compartment. **(A)** Gating strategy used to identify the senescent/clonal expanded and exhausted CD8^+^ T cells in the CAR^+^ compartment. Pie charts report the percentage of exhausted and senescent/clonal expanded CAR^+^CD8^+^ T cells at **(B)** pre-PBZ and **(C)** D+21 and D+42 after PBZ. **(D)** Gating strategy used to identify the senescent/clonal expanded and exhausted CD8^+^ T cells in the non-CAR CD8^+^ T cell compartment. Pie charts report the percentage of **(E)** exhausted and senescent/clonal expanded CAR^-^CD8^+^ T cells at pre-PBZ, **(F)** exhausted and **(G)** senescent/clonal expanded CAR^-^CD8^+^ T cells at D+21 and D+42 after PBZ. **(H)** Identification of pathogenetic genetic alterations in the ccfDNA at LD (blu bars, n=16) and at day +42 (orange bars, n=18) and relative VAF. **(I)** Dot plots display CD3^+^ and CD3^+^CD19 CAR^+^ T cell populations before and after PBZ.

Before PBZ treatment, the entire compartment of circulating CAR^+^ T cells was set up by a population of terminally differentiated (CD45RA^+^ CD62L^-^), senescent (CD28^-^CD57^+^), exhausted (PD-1^+^) CD8^+^ cells (**Figures 2A, B**). Notably, PBZ treatment did not modify the percentages of neither exhausted nor senescent CAR^+^T cells (**Figure 2C**). In the non-CAR cell compartment, a remarkably high percentage of CD8^+^ T cells showed exhausted (69%) and senescent (40%) phenotype at pre-PBZ time point (**Figures 2D, E**). At variance to CAR^+^ cells, PBZ treatment caused a decrease in the percentage of non-CAR CD8^+^PD-1^+^ T cells at day+21, that was paralleled by the resolution of clinical symptoms. However, the percentage of non-CAR CD8^+^PD-1^+^ T cells increased again at day+42 (**Figure 2F**), when PET/CT scans showed signs of disease progression (see **Figure 1E**). Noteworthy, the percentage of senescent non-CAR CD8^+^ T cells increased upon PBZ administration (**Figure 2G**). Finally, out of a total of 18 mutations detected in the circulating cell free (ccf) DNA at day +42, at least 4 mutations (in *KDR*, *TP53*, *ARID1A* and *KMT2C* loci) were almost undetectable in the ccfDNA at the LD timepoint, when a total of 16 mutations were detectable (**Figure 2H**).

## Discussion

Here, we report on a patient affected by refractory DLBCL who was resistant to CAR T-cell therapy and to two cycles post CAR-T of PZB due to the evolution into a B-cell Hodgkin-like Lymphoma. In fact, PZB is envisaged as a potential tool to reinvigorate exhausted CAR-T cells in lymphoma patients (7), as exhaustion is a reversible mechanism (8). Instead, PBZ is not able to reinvigorate exhausted CAR^+^ T cells in this patient, as also suggested by the limited and transient expansion of CAR^+^ T cells upon PBZ treatment (see dot plots in **Figure 2I**). We hypothesize that the lack of PBZ efficacy is due to the senescent phenotype of the CAR^+^ T cells population. Indeed, by definition, senescence confers an irreversible loss of proliferative capacity (8). Thus, we propose that the assessment of exhaustion markers in CAR-T cells should be accompanied by the evaluation of T cell senescence features, in order to better estimate the potential role of PBZ in post-CAR-T failure and rescue.

Regarding the non-CAR CD8^+^ T cell compartment, PBZ transiently induced, on the one hand a decrease in the percentage of exhausted CD8^+^ T cells, on the other hand an increase in senescent CD8^+^ T cells. Again, we underpin that the onset of senescence is an irreversible phenomenon that is likely to contribute to the creation of an immune landscape with poor antitumor activity (8). Even the loss of CD19 antigen by tumor cells, likely due to drug-induced clonal selection, is expected to hinder the proliferation potential of CAR^+^ T cells. As an index of clonal evolution, we observed changes in the mutational landscape of tumor-derived ccfDNA after PBZ. In particular, *ARID1A* gene mutations were observed at day+42, concomitantly with the onset of disease progression. Notably, *ARID1A* gene mutations occur in about 10% of DLBCL and 7% of HL (9), and have been associated with resistance to PBZ, by tampering with interferon pathway (10). We conclude that the combined analysis of the exhausted/senescent immune profile in CAR-T patients, together with the assessment of genetic alterations in ccfDNA may provide a realistic snapshot of disease evolution and chance to treatment response.

## Data availability statement

The sequencing data presented in this study have been submitted to the Sequence Read Archive (SRA) online repository. The samples can be retrieved at https://www.ncbi.nlm.nih.gov/sra using the accession number SRR17520182 (for time point LD) and SRR17520181 (for time point +42 after PBZ).

## Ethics statement

This study was reviewed and approved by Ethical Committee AVEC of Bologna, Italy. The patients/participants provided their written informed consent to participate in this study. Written informed consent was obtained from the individual(s) for the publication of any potentially identifiable images or data included in this article.

## Author contributions

Contribution: SD, BC, FBo and PZ initiated the project, designed the research, and wrote the paper with input from other authors. SD, NL, GS, SB, MF, MB performed correlative laboratory studies, assisted with data analysis. ED, BS, LZ performed CAR T-cells thawing and processing in the tissue establishment. BC, GL, MD, FBa, AP, AA, PB, CB provided clinical data and clinical care for the patient, interpreted the data. All authors contributed to the article and approved the submitted version.

## Funding

The work reported in this publication was funded by the Italian Ministry of Health, RC-2022-2773291. Project: Identificazione di biomarcatori di risposta clinica e di insorgenza di complicanze in pazienti ematologici sottoposti a terapia CAR T.

## Acknowledgments

The authors thanks AIL Bologna ODV for the support of the Immunobiology of transplant and cellular therapies, IRCCS AOU di Bologna, Bologna, Italy.

## Conflict of interest

PZ: scientific advisory boards: Secura Bio, Celltrion, Gilead, Janssen-Cilag, BMS, Servier, Sandoz, MSD, TG Therap., Takeda, Roche, EUSA Pharma, Kiowa Kirin, Novartis, ADC Therap., Incyte, Beigene; consultancy: EUSA Pharma, MSD, Novartis; speaker’s bureau: Celltrion, Gilead, Janssen-Cilag, BMS, Servier, MSD, TG Therap., Takeda, Roche, EUSA Pharma, Kiowa Kirin, Novartis, Incyte, Beigene. FB: scientific advisory boards and speaker fees: NEOVII, NOVARTIS, KITE, GILEAD, PFIZER, CELGENE, MSD. MB: Research Grant from NEOVII.

The remaining authors declare that the research was conducted in the absence of any commercial or financial relationships that could be construed as a potential conflict of interest.

## Publisher’s note

All claims expressed in this article are solely those of the authors and do not necessarily represent those of their affiliated organizations, or those of the publisher, the editors and the reviewers. Any product that may be evaluated in this article, or claim that may be made by its manufacturer, is not guaranteed or endorsed by the publisher.
